# Life Cycle Assessment and Performance Evaluation of Self-Compacting Concrete Incorporating Waste Marble Powder and Aggregates

**DOI:** 10.3390/ma18132982

**Published:** 2025-06-24

**Authors:** Masoud Ahmadi, Erfan Abdollahzadeh, Mohammad Kashfi, Behnoosh Khataei, Marzie Razavi

**Affiliations:** 1 Department of Civil Engineering, Faculty of Engineering, Ayatollah Boroujerdi University, Boroujerd 6919969737, Iran; erfan.abdolahzadeh93@gmail.com; 2 Faculty of Earth Sciences Engineering, Arak University of Technology, Arak 3818146763, Iran; b.khataei@arakut.ac.ir; 3 Department of Mechanical Engineering, Faculty of Engineering, Ayatollah Boroujerdi University, Boroujerd 6919969737, Iran; m.kashfi@abru.ac.ir; 4 Department of Civil Engineering, Tafresh University, Tafresh 3951879611, Iran; m.razavi@tafreshu.ac.ir

**Keywords:** life cycle assessment, SCC, waste marble, durability, sustainability

## Abstract

This study systematically investigates the utilization of marble industry waste—waste marble powder (WMP) as partial cement replacement and waste marble aggregates (WMA) as partial fine aggregate replacement—in self-compacting concrete (SCC). A detailed experimental program evaluated the effects of various replacement levels (5%, 10%, and 20% for WMP; 20%, 30%, and 40% for WMA) on compressive strength and durability, particularly resistance to aggressive sulfuric acid environments. Results indicated that a 5% WMP replacement increased compressive strength by 4.9%, attributed primarily to the filler effect, whereas higher levels (10–20%) led to strength reductions due to limited pozzolanic activity and cement dilution. In contrast, WMA replacement consistently enhanced strength (maximum increase of 11.5% at 30% substitution) due to improved particle packing and aggregate-paste interface densification. Durability tests revealed significantly reduced compressive strength losses and mass loss in marble-containing mixtures compared to control samples, with optimal acid resistance observed at 20% WMP and 40% WMA replacements. A comprehensive life cycle assessment demonstrated notable reductions in environmental impacts, including up to 20% decreases in Global Warming Potential (GWP) at 20% WMP replacement. A desirability-based eco-cost-mechanical optimization—simultaneously integrating mechanical strength, environmental indicators, and production cost—identified the 10% WMP substitution mix as the most sustainable option, achieving optimal balance among key performance criteria. These findings underscore the significant potential for marble waste reuse in SCC, promoting environmental sustainability, resource efficiency, and improved concrete durability in chemically aggressive environments.

## 1. Introduction

Concrete is the most consumed building material worldwide, primarily due to rapid urban development and extensive infrastructure projects [1,2,3]. Nonetheless, its production process contributes heavily to environmental degradation, largely from the intensive use of cement and extraction of natural aggregates. Cement production alone is responsible for nearly 5–8% of the total global carbon dioxide emissions [4,5,6,7,8]. Furthermore, the extraction of natural aggregates contributes to habitat destruction, landscape degradation, and resource depletion, raising concerns about the long-term environmental sustainability of concrete production [9]. Consequently, the pursuit of environmentally responsible alternatives has gained momentum, especially through the incorporation of industrial by-products, such as marble waste, into concrete formulations [10,11,12,13,14,15].

The marble industry generates considerable quantities of waste, including dust, slurry, and stone fragments, during both the extraction and processing phases [16,17,18,19]. The disposal of this waste poses challenges such as landfill overburden, environmental pollution, and increased disposal costs. In response, researchers have explored the potential for incorporating marble waste as a replacement for traditional concrete components, including natural aggregates and cement, to reduce environmental impact and enhance concrete performance [20,21,22].

A growing number of investigations have examined the use of recycled marble aggregates (RMAs) as partial or full replacements for natural aggregates, revealing encouraging results in terms of strength and durability. For example, Binici et al. [23] reported that concrete blends using marble and granite waste, along with ground blast furnace slag, achieved improved compressive strength (up to 62.1 MPa) and demonstrated superior resistance to physical and chemical deterioration mechanisms. Similarly, Hebhoub et al. [24] evaluated the substitution of natural aggregates with waste marble aggregates (WMAs) in concrete production, considering replacement rates of 25%, 50%, 75%, and 100%. The study found that partial replacements (25–75%) led to enhanced compressive and tensile strengths, whereas a 100% replacement resulted in strength reduction. This investigation highlighted that, when appropriately used, WMAs could improve mechanical performance, further supporting their application as sustainable alternatives in concrete production. Silva et al. [25] investigated the mechanical properties of structural concrete incorporating fine aggregates derived from marble quarrying waste, with replacement levels of 0%, 20%, 50%, and 100%. Although a slight reduction in strength and durability was observed with increasing waste marble (WM) content, the performance remained within acceptable limits for structural applications, emphasizing its potential as a sustainable substitute for natural aggregates. Uygunoglu et al. [26] examined the incorporation of WM and recycled aggregate (RA) as full replacements for natural limestone aggregate in self-compacting concrete (SCC), aiming to enhance environmental sustainability. Their study demonstrated that the use of WM improved the fresh properties of SCC, including flowability and segregation resistance, without significantly compromising mechanical performance. RA exhibited acceptable strength and workability, although with a slightly reduced modulus of elasticity and compressive strength at higher water-binder ratios. This research underscores the viability of substituting conventional aggregates with industrial by-products in SCC production, contributing to resource conservation and waste reduction in the construction industry. Vardhan et al. [27] identified 40% marble sand replacement as an optimal balance between enhanced compressive strength and reduced shrinkage, also developing a shrinkage prediction model. In another example, Benjeddou et al. [28] investigated the development of lightweight concrete incorporating marble waste sand (MWS) and expanded perlite aggregate (EPA). Their findings demonstrated that substituting MWS with more than 20% EPA significantly reduced concrete density, improved thermal insulation, and increased sound reduction capacity, positioning the mixture as a sustainable solution for energy-efficient construction.

The disposal of waste marble powder (WMP), another by-product of the marble industry, has also garnered attention due to its adverse environmental impacts, prompting researchers to investigate its use as a partial replacement for cement or sand in concrete [29]. Studies indicate that replacing sand with marble dust (up to 15%) can improve mechanical performance, particularly at around 10% content [30]. Ashish [14] observed that a concrete mixture with 10% metakaolin and 15% WMP exhibited enhanced strength properties relative to both control mixes and those containing silica fume. Long-term durability assessments involving cement replacement with dried marble powder slurry (10–25%) demonstrated improved structural performance for substitution levels up to 15% after one year in severe exposure environments [31]. Lezzerini et al. [32] noted a reduction in compressive strength with higher WMP content but still reported values around 43 MPa at 28 days, suitable for structural use. They attributed the decline to WMP’s limited pozzolanic activity and inert filler characteristics. Nonetheless, parameters like density and water absorption remained within acceptable ranges. Jia et al. [33] achieved strength optimization at 5–10% replacement using modified WMP, though higher levels led to performance losses due to dilution of cement. Rana et al. [34] found that a 10% marble slurry replacement provided the most favorable results in terms of strength, permeability, and resistance to aggressive agents. Altogether, these studies suggest that WMP can serve as a sustainable cement replacement, particularly when used in synergy with SCMs or activated through physical or chemical treatments, offering a path toward greener concrete solutions.

Recent developments in numerical modeling—particularly through cohesive crack models and continuum damage mechanics—have enhanced our understanding of cracking and degradation mechanisms in concrete. Notably, De Maio et al. [35] applied an improved cohesive zone model to assess the degradation of dynamic properties in plain concrete under mixed-mode fracture conditions, while Turcry et al. [36] combined experimental and numerical approaches to investigate shrinkage-induced cracking in SCC. Although the present study is experimental in nature, integrating such numerical frameworks with durability and environmental performance assessments offers a promising avenue for future research, particularly for predicting long-term performance in aggressive environments.

Although prior studies [37,38,39,40,41] have examined the effects of marble waste on specific properties of SCC, the novelty of the present research lies in its comprehensive and integrated methodological framework. This study simultaneously assesses mechanical performance, sulfuric acid resistance as a key durability parameter, environmental impact via detailed life cycle assessment (LCA), and economic viability. Moreover, a desirability-based multi-objective optimization approach is applied to determine the optimal mix proportions, providing a practical pathway for producing cost-effective and environmentally sustainable SCC incorporating marble industry waste. By integrating economic analysis alongside environmental and mechanical assessments, this research ensures both technical feasibility and real-world applicability, thereby supporting informed decision-making in sustainable concrete production.

## 2. Research Significance

Although numerous studies have investigated the individual use of WMP as a cement substitute and WMA as a fine aggregate replacement in concrete, key knowledge gaps remain. While replacement levels of 5–15% for WMP and 10–40% for WMA have demonstrated improvements in mechanical performance and environmental impact, there is still inconsistency in identifying the most effective substitution rates, particularly under aggressive exposure conditions. Moreover, most prior research has evaluated WMP and WMA independently, with limited focus on their combined effects, especially under chemically aggressive environments. Of particular concern is sulfuric acid attack, which is highly relevant due to its frequent occurrence in sewer and wastewater systems where biogenic oxidation of hydrogen sulfide generates sulfuric acid. This results in extremely low pH environments that impose severe chemical degradation on concrete. Consequently, there is a pressing need for comprehensive studies that examine the combined incorporation of WMP and WMA in SCC, particularly under exposure conditions representative of sewage treatment plants and industrial infrastructures.

In response to this research need, the current study is designed to thoroughly investigate the mechanical behavior and acid resistance of SCC containing WMP and WMA as partial replacements for cement and fine aggregate, respectively. Particular emphasis is placed on evaluating compressive strength development and material degradation following prolonged exposure to sulfuric acid. The research aims to pinpoint the optimal replacement percentages that yield a favorable balance between mechanical strength and chemical durability. Moreover, by evaluating mass loss and compressive strength retention, the study provides insight into the degradation mechanisms in acid-exposed concrete.

Beyond technical performance, this work incorporates a sustainability perspective, recognizing that replacing virgin materials with marble waste can significantly reduce both the environmental footprint and production costs of concrete. Accordingly, the integration of mechanical, environmental, and economic assessments supports a holistic evaluation, enabling the identification of optimal mix designs for sustainable and high-performance SCC applications.

## 3. Materials and Mix Design

### 3.1. Materials Characteristics

The composition of the SCC mixtures evaluated in this study consisted of ordinary Portland cement (OPC), WMP, natural crushed fine aggregates, WMA, a high-range water-reducing admixture based on polycarboxylate chemistry, and potable water. These materials were chosen considering their local availability, environmental benefits, and their ability to satisfy the fresh and hardened property requirements of SCC formulations.

#### 3.1.1. Binder

The cementitious system included OPC as the principal binder, partially substituted by WMP. OPC was selected for its proven mechanical performance and compliance with ASTM C150 Type I specifications [42]. The WMP, sourced from marble processing operations, was introduced as a sustainable alternative to reduce cement consumption and carbon emissions. The elemental composition of both OPC and WMP was determined via X-ray fluorescence spectroscopy, with results presented in Table 1 confirming their compatibility with cement-based applications.

#### 3.1.2. Aggregates

Two types of fine aggregates were used: naturally crushed sand and recycled WMA. Particle size analysis was performed following ASTM C136 [43] to ensure compliance with SCC grading requirements. The natural sand was sieved to retain particles passing through a 4.75 mm sieve but retained above the 0.075 mm threshold to maintain gradation consistency. WMAs were obtained by crushing marble waste, sieved through a 17 mm mesh, and collected between 2.38 mm and 17 mm to meet gradation limits suitable for SCC.

#### 3.1.3. Chemical Admixture

A polycarboxylate-based superplasticizer was utilized to enhance flow characteristics and ensure sufficient workability for SCC mixtures. The admixture met the requirements of ASTM C494 [44] Type F and was incorporated in different dosages to optimize the mix’s self-compacting properties while ensuring uniform dispersion of cementitious materials.

#### 3.1.4. Water

Potable water, free from impurities, was used in accordance with ASTM C1602 [45] to ensure adequate hydration and proper development of mechanical properties. The water-to-binder ratio was carefully controlled to achieve the desired consistency and minimize excessive bleeding or segregation.

### 3.2. Mix Proportions

The experimental mix designs in this study were developed to investigate the influence of substituting cement with WMP and natural fine aggregates with WMA on the performance of SCC. A total of seven concrete mixes were produced. The control mixture (CM) contained 100% OPC and 100% natural sand as fine aggregate, with a fixed water-to-binder (w/b) ratio of 0.32 applied uniformly across all mixes. To evaluate the effect of incorporating WMP as a cement replacement, three mixes—MP5, MP10, and MP20—were formulated by replacing 5%, 10%, and 20% of OPC with WMP, respectively, while retaining natural sand as the sole fine aggregate. For the aggregate replacement study, mixes labeled MA20, MA30, and MA40 were prepared by substituting 20%, 30%, and 40% of natural sand with WMA, respectively, without altering the OPC content in the binder. Table 2 provides the specific proportions for each mix, detailing the percentages of OPC and WMP in the binder, as well as the relative amounts of WMA and sand in the fine aggregate portion. In this study, WMA was used as a partial replacement for natural sand on an equal-mass basis. This method was selected to represent a pragmatic and widely practiced substitution approach in concrete technology. While minor deviations in volume fractions may arise due to density differences between WMA and natural sand, the mix designs were experimentally validated to meet SCC workability criteria, ensuring consistent performance conditions across all mixtures.

It is important to note that the mix design for CM, MA20, and MA40 was previously investigated by the authors in an earlier study [19]. However, all experimental testing, durability evaluations, life cycle assessments, cost analyses, and optimization conducted in the present study are entirely original and were performed specifically for this research.

### 3.3. Test Program

#### 3.3.1. Compressive Strength Test

The compressive strength performance of the concrete mixes listed in Table 2 was evaluated using a standard testing protocol. For each of the seven SCC mix types, three cube specimens measuring 100 mm on each side (100 × 100 × 100 mm) were cast, totaling 21 specimens. Following an initial curing period of 24 h, the molds were removed, and the samples were transferred to a water-curing tank, where they were maintained for 28 days under controlled conditions. Upon completion of the curing period, compressive strength measurements were performed using a hydraulic testing machine with a maximum capacity of 200 tons. The procedure adhered to BS EN 12390-3 [46], ensuring consistency and accuracy in accordance with accepted international testing standards.

#### 3.3.2. Sulfuric Acid Attack Test

The durability of SCC mixtures under acidic conditions was evaluated following the guidelines set forth in ASTM C267 [47]. This method involved immersing 100 mm concrete cubes in a sulfuric acid solution to simulate a highly aggressive chemical environment. After completing a 28-day water curing phase, the specimens were taken out of the curing tank and transferred to a sulfuric acid bath maintained at a pH level of 1. Since ongoing chemical interaction between sulfuric acid and concrete tends to gradually elevate the pH of the solution, periodic replenishment with diluted acid was performed to maintain the desired pH throughout the exposure period. The acid solution was stirred three times per week to maintain uniformity, and pH was continuously monitored using a calibrated digital pH meter to ensure precision. Adjustments were carried out as needed to stabilize the acidity level.

**Figure 1:**
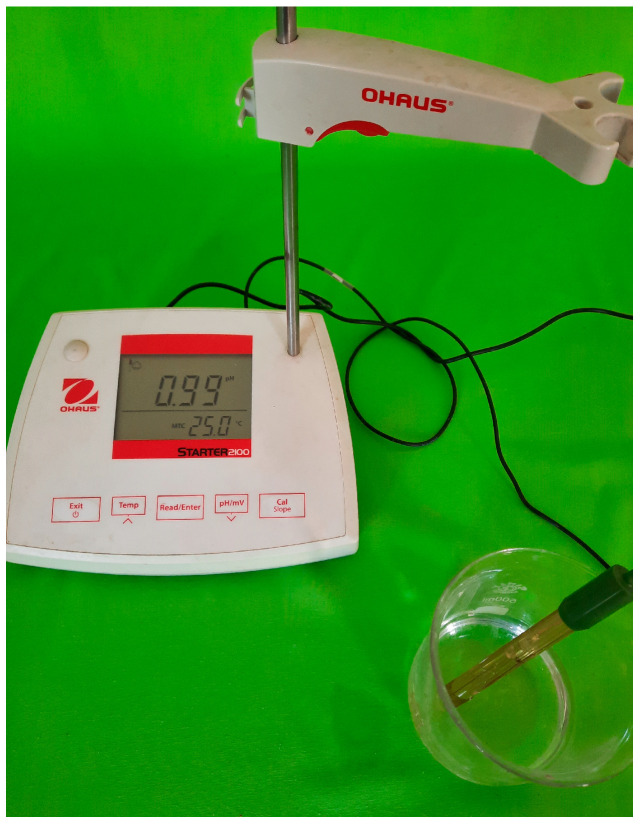
pH testing of sulfuric acid solution.

**Figure 2:**
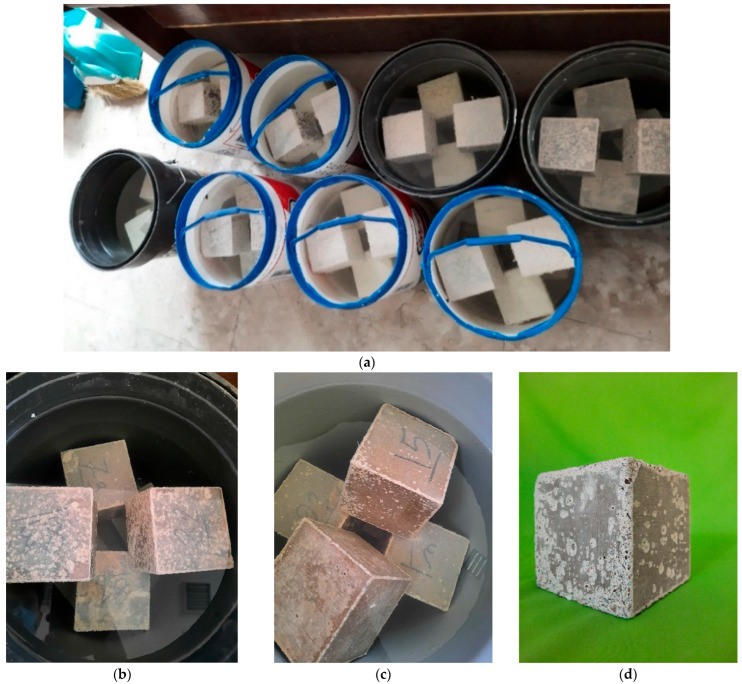
Placement of specimens in sulfuric acid solution. (**a**) Overview of acid immersion containers with concrete cubes; (**b**) Close-up view of submerged specimens after exposure; (**c**) Surface condition of specimens following sulfuric acid exposure; (**d**) Visible surface degradation and gypsum formation on a specimen after extended immersion.

For each of the seven SCC mixtures, six specimens were prepared, resulting in a total of 42 samples subjected to acid exposure. These specimens were tested at two intervals—28 days and 63 days—to assess progressive degradation. Three samples from each mix were evaluated at each time point. At the end of each exposure period, measurements were taken for both weight loss and residual compressive strength to quantify the extent of deterioration. Figure 1 and Figure 2 depict the pH monitoring procedure and the acid immersion setup. Specimens were not visibly labeled during acid exposure, as labels could deteriorate or interfere with the chemical reactions in the highly acidic solution. Instead, sample identification was maintained through pre-exposure coding and tracking.

## 4. Results and Discussion

### 4.1. Mechanical and Durability Properties

#### 4.1.1. Compressive Strength

**Figure 3:**
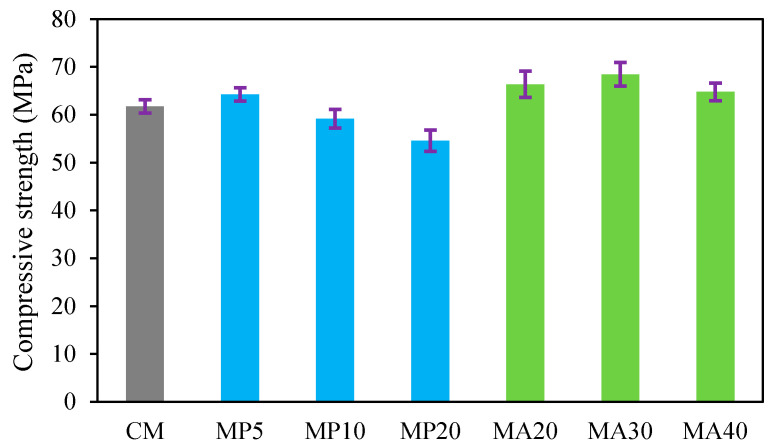
Effect of WMP and WMA on the compressive strength.

The 28-day compressive strength results of the SCC mixtures incorporating WMP as a partial cement replacement (MP5, MP10, MP20) and WMA as a partial fine aggregate replacement (MA20, MA30, MA40) are presented in Figure 3. The measured compressive strengths ranged from 54 MPa (MP20) to 68 MPa (MA30), compared to 61 MPa for the CM. The relative compressive strengths, expressed as a ratio to CM, varied from 0.885 (MP20) to 1.114 (MA30). The MA series (MA20, MA30, MA40) generally outperformed the CM, whereas the MP series (MP5, MP10, MP20) exhibited a decline in strength with increasing replacement levels, except for MP5, which showed a slight increase compared to the CM. These trends are graphically illustrated in Figure 3, with compressive strength on the left vertical axis and relative compressive strength on the right vertical axis.

##### Effect of WMP as OPC Replacement

Among the mixes incorporating waste marble powder (WMP) as a partial replacement for OPC, MP5 exhibited a compressive strength increase of approximately 4.92% compared to the CM. In contrast, MP10 and MP20 experienced strength reductions of 3.28% and 11.47%, respectively. These results are in agreement with previous studies [14,29,30], which suggest that low WMP replacement levels (generally up to 5–10%) can enhance strength due to a beneficial filler effect. This effect stems from the ability of ultrafine WMP particles to occupy microvoids in the cement paste, thereby increasing packing density and decreasing porosity. This densification contributes to improved particle interlocking, which, as reported by [30,31], promotes higher compressive strength in mixes with modest WMP content.

However, increasing WMP content to 10% or 20% led to noticeable reductions in compressive strength. This decline is primarily due to WMP’s inert chemical nature and its lack of pozzolanic activity. Unlike reactive mineral admixtures such as silica fume or fly ash, WMP is composed mainly of calcium carbonate (CaCO_3_), which does not contribute to the formation of calcium silicate hydrate (C-S-H)—the principal binder phase generated during cement hydration. Findings from [30] reinforce that WMP acts primarily as a non-reactive filler without engaging in chemical reactions that enhance strength development.

Further supporting this trend, Ashish [14] and Lezzerini et al. [32] reported that at higher replacement levels (beyond 10–15%), WMP may increase overall porosity and reduce the cohesiveness of the cementitious matrix. This weakens the interfacial transition zone between the paste and aggregates, which is critical to mechanical strength. The observed strength reductions in MP10 and MP20 are, therefore, consistent with these mechanisms and underscore the limitations of high-level WMP incorporation in structural concrete.

##### Effect of WMA as Fine Aggregate Replacement

In contrast to the mixtures incorporating WMP, partial substitution of natural fine aggregates with WMA in the MA20, MA30, and MA40 mixes consistently enhanced compressive strength, with the highest increase of 11.47% recorded for MA30. When the WMA replacement level was increased to 40% (MA40), compressive strength continued to improve, reaching 64 MPa (+4.9% relative to the CM). However, the rate of improvement diminished compared to the previous two replacement levels, suggesting that while WMA positively contributes to strength development, an upper threshold exists beyond which the benefits become less pronounced.

Unlike conventional fine aggregates, the WMA used in this study was coarser, retained between 2.38 mm and 17 mm while still meeting ASTM C136 [43] gradation requirements for SCC applications. The increase in compressive strength observed at all replacement levels can be attributed to optimized particle size distribution, which enhances packing density by improving aggregate gradation and reducing interstitial voids. This effect refines the granular skeleton, leading to a denser microstructure and improved mechanical interlocking between aggregates.

These findings align with Hebhoub et al. [24], who reported that a well-graded WMA fraction enhances compressive strength by increasing matrix densification. Furthermore, Silva et al. [25] highlighted that the inclusion of WMA improves microstructural densification, which may contribute to enhanced paste cohesion through potential secondary chemical interactions with cement hydration products. However, the dominant mechanism behind the strength improvement remains the enhanced packing density and ITZ performance rather than chemical reactivity. The combined effects of improved particle packing and potential secondary chemical interactions explain the consistent strength gain across all replacement levels.

Although compressive strength continued to improve at 40% replacement (MA40), the rate of increase declined compared to MA20 and MA30. This trend suggests that while WMA contributes positively to strength development, the benefits become less pronounced beyond an optimal replacement threshold. Several factors explain this diminishing rate of improvement. One key factor is the gradual saturation of packing efficiency. The enhanced packing density and optimized aggregate gradation observed at 20% and 30% replacement levels had already significantly refined the granular skeleton of the SCC matrix. Binici et al. [23] noted that beyond a certain replacement threshold, further increases in WMA content may not yield proportional strength gains as the aggregate packing effect reaches a saturation point, limiting additional improvements in compressive strength. Another contributing factor is the reduction in aggregate-paste bonding efficiency. While moderate WMA incorporation (20–30%) improved microstructural densification, higher replacement levels introduced more ITZs due to the coarser size and smoother surface texture of WMA compared to natural fine aggregate. Uygunoglu et al. [26] observed that excessive WMA may reduce ITZ performance due to its flaky shape, which weakens aggregate-paste adhesion and leads to lower splitting tensile strength. Furthermore, higher WMA replacement levels may lead to an increase in porosity, offsetting the benefits of matrix densification. While moderate WMA contents help improve microstructural compaction, excessive amounts may introduce localized voids due to minor inconsistencies in packing efficiency at higher replacement levels. Hebhoub et al. [24] found that higher WMA fractions can increase interparticle voids, potentially leading to a marginal decline in mechanical performance beyond the optimal range.

#### 4.1.2. Resistance to Sulfuric Acid Attack

The durability performance of SCC mixes containing WMP and WMA was evaluated under highly acidic conditions by tracking compressive strength retention and mass loss after exposure to a sulfuric acid solution maintained at pH 1. The specimens were examined after immersion periods of 28 and 63 days.

##### Compressive Strength Loss

**Figure 4:**
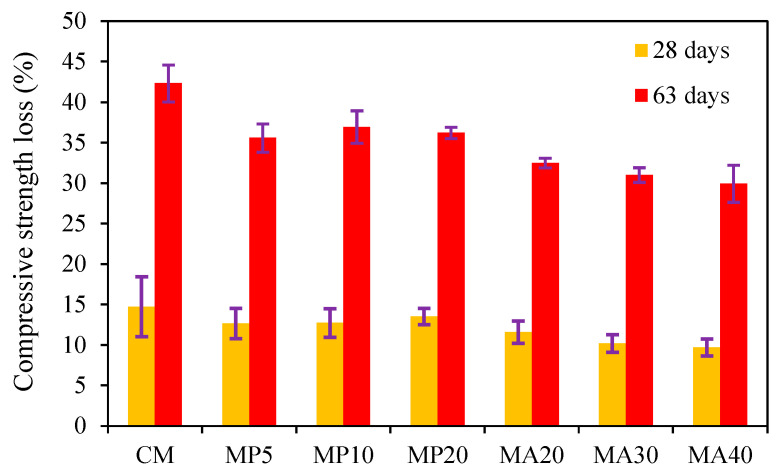
Compressive strength loss for specimens after immersion in solution for 28 and 63 days.

The compressive strength loss of SCC samples exposed to the sulfuric acid solution (pH = 1) for 28 and 63 days is presented in Figure 4. The results reveal that all marble-containing mixtures (MP5, MP10, MP20, MA20, MA30, MA40) exhibited lower compressive strength loss compared to the CM across both exposure durations. After 28 days, the CM experienced a strength loss of 14.75%, whereas the strength losses in the MP and MA series ranged from 9.7% to 13.52%. After 63 days of acid exposure, the CM showed the highest strength loss (42.32%), while the marble-containing samples exhibited reduced losses, ranging from 29.94% to 36.94%.

Among the mixtures, MP5 demonstrated the lowest strength loss in the marble powder series (12.68% at 28 days and 35.6% at 63 days), indicating notable acid resistance at lower powder replacement levels. Similarly, MA40 exhibited the best performance among the marble aggregate series, with strength losses of 9.7% at 28 days and 29.94% at 63 days. These findings suggest that a lower replacement level of marble powder and a higher replacement level of marble aggregates enhance sulfuric acid resistance.

The improved acid resistance of marble-based SCC can be attributed to several factors. First, the filler effect of WMP enhances the packing density of the matrix, reducing porosity and hindering acid ingress, consistent with the findings of Khodabakhshian et al. [48] and Selim et al. [49]. Second, the chemical composition of marble materials, predominantly calcium carbonate (CaCO_3_), can react with sulfuric acid to form a superficial gypsum layer (CaSO_4_·2H_2_O). Although gypsum formation is a widely reported mechanism in sulfuric acid attack, its presence in this study was inferred from performance-based deterioration trends. This layer can initially act as a physical barrier, slowing further acid penetration and delaying deterioration, as observed by [50,51]. A similar protective gypsum formation mechanism was also identified during a sulfate attack in SCC containing marble and tile waste by Tennich et al. [52].

However, it is important to note that this protective effect is temporary. Prolonged acid exposure leads to the accumulation and thickening of the gypsum layer, which can induce volumetric expansion, surface spalling, and microcracking, ultimately accelerating strength degradation. This long-term deterioration mechanism under aggressive sulfuric acid exposure has been highlighted in the works of Selim et al. [49] and Xiao et al. [51]. Overall, the reduced compressive strength losses in MP20 and MA40 demonstrate that increasing the replacement levels of both WMP and WMA enhances the acid resistance of SCC. This supports the potential application of marble-based SCC in aggressive environments, such as sewage treatment plants and industrial facilities exposed to acidic solutions.

##### Mass Loss

**Figure 5:**
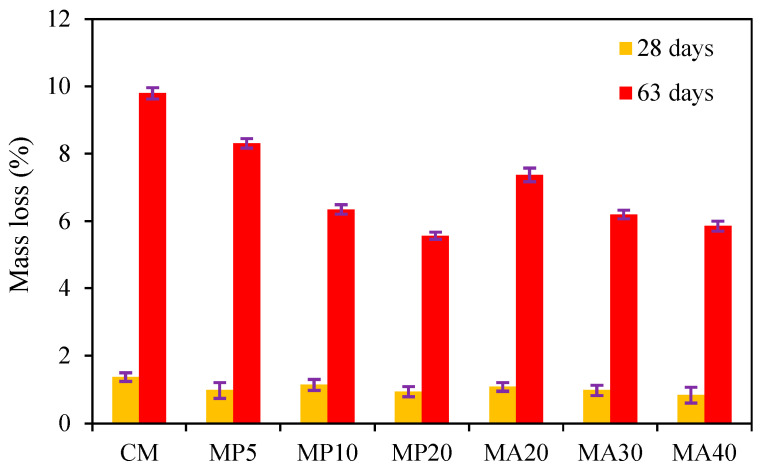
Mass loss for specimens after immersion in solution for 28 and 63 days.

The mass loss results of the SCC specimens immersed in sulfuric acid solution (pH = 1) for 28 and 63 days are depicted in Figure 5. The data indicate that all mixtures incorporating marble powder (MP5, MP10, MP20) and marble aggregate (MA20, MA30, MA40) experienced lower mass loss compared to the CM over both exposure durations. After 28 days of acid immersion, the CM exhibited a mass loss of 1.37%, while the MP series ranged from 0.94% to 1.14% and the MA series from 0.84% to 1.08%.

Upon prolonged exposure (63 days), the CM recorded a mass loss of 9.8%, whereas the MP series exhibited losses between 5.57% and 8.31% and the MA series between 5.86% and 7.38%. The MA40 mixture achieved the lowest mass loss at both 28 days (0.84%) and 63 days (5.86%), demonstrating superior resistance to acid-induced deterioration. Similarly, within the MP series, MP20 exhibited the best performance, with mass losses of 0.94% and 5.57% at 28 and 63 days, respectively.

These results indicate that higher replacement levels of both WMP and WMA significantly reduce mass loss under sulfuric acid exposure, affirming the positive influence of marble waste on SCC durability in aggressive acidic environments. The improved acid resistance and reduced mass loss in marble-containing SCC mixtures can be attributed to several complementary mechanisms. First, the filler effect of WMP contributes to matrix densification and reduced porosity, limiting acid penetration and reducing the leaching of hydration products, as reported by [48,49]. Second, the chemical stability and inert nature of marble aggregates reduce the dissolution of aggregate particles under acid attack, enhancing overall material integrity. Third, the reaction between calcium carbonate (CaCO_3_), the primary component of marble waste, and sulfuric acid (H_2_SO_4_) results in the formation of a gypsum layer (CaSO_4_·2H_2_O). This layer temporarily retards acid ingress and mass loss, as noted by [50,51]. However, long-term gypsum accumulation may lead to surface scaling and mass detachment, particularly in weaker matrices.

The superior performance of the MA40 and MP20 mixtures suggests that increasing the dosage of marble waste enhances the acid resistance of SCC, minimizing both strength degradation and mass loss. These findings support the viability of marble waste as a sustainable alternative in SCC formulations intended for acidic environments, such as sewage systems, wastewater treatment plants, and industrial facilities.

### 4.2. Life Cycle Assessment

A comprehensive life cycle assessment (LCA) was conducted to quantify the environmental impacts of the various SCC mixtures developed in this study. This analysis considered six key environmental indicators: Ozone Depletion Potential (ODP), Acidification Potential (AP), Eutrophication Potential (EP), Photochemical Ozone Creation Potential (POCP), Global Warming Potential (GWP), and Fossil Fuel Depletion Potential (FP). The significance of each indicator is summarized below.

#### 4.2.1. Environmental Indicators

Ozone Depletion Potential: ODP evaluates the impact of substances on ozone depletion. Ozone protects the Earth from harmful UV radiation in the stratosphere. Substances with high ODP values can break down ozone molecules, leading to ozone layer thinning and increased UV exposure. ODP offers vital information for scientists and industries to regulate ozone-depleting substances. Recognizing the significance of ODP, prompts proactive measures to combat ozone depletion, endorse sustainability, and support global atmospheric protection efforts.

Acidification Potential: AP is one of the environmental indicators to assess the impact of acidic substances on the environment. It helps measure substances’ potential to lower soil, water, and air pH levels, harming ecosystems. Quantifying AP aids in prioritizing the reduction of emissions causing acid rain and environmental harm. Monitoring AP identifies acidic emission sources for targeted reduction strategies. AP is valuable for environmental and sustainability assessments, comparing products and processes for sustainability.

Eutrophication Potential: EP indicates the risk level of nutrient enrichment in water bodies and the subsequent harmful effects on ecosystems. By evaluating the EP of different substances, various industries can prioritize using environmentally friendly products and practices to reduce nutrient runoff and mitigate the risks of eutrophication.

Photochemical Ozone Creation Potential: POCP is crucial in assessing substances’ contribution to ground-level ozone formation. It determines emissions’ ability to react with sunlight and pollutants, producing ozone with harmful effects on health and air quality. POCP is key in evaluating air pollution control effectiveness and guiding tailored interventions for improved air quality.

Global Warming Potential: GWP is crucial for evaluating the impact of greenhouse gas emissions on climate change. Industries use GWP to reduce emissions and transition to low-carbon alternatives. Integrating GWP into life cycle assessments and carbon footprint analyses enhances the understanding of products’ climate impact. Calculating GWP helps companies minimize their carbon footprint for climate resilience and sustainability.

Fossil Fuel Depletion Potential: FP quantifies activities’ potential to emit fossil fuel reserves, leading to resource scarcity and environmental damage. It guides the sustainability of energy systems and the transition to cleaner energy sources. By integrating FP into energy planning and policy development, authorities can promote clean energy adoption, reduce emissions, and enhance energy security. Besides, incorporating FP into life cycle assessments and sustainability evaluations provides a comprehensive understanding of the environmental impact of energy, supporting optimizing energy consumption and advancing sustainability.

In the present study, to evaluate the sustainability of the concretes prepared with waste marble aggregate and marble powder, the environmental indicator (EI) related to the production of 1 m^3^ of concrete has been calculated according to Equation (1).

(1) $$EI=\sum_{i}m_{i}\times {EI}_{i}$$

where: *m_i_* = mass (in kg) of inventory flow *i*, and *EI_i_* = amount of the environmental indicator including ODP, AP, EP, POCP, GWP, FP (presented in Table 3 as life cycle inventory of raw materials) in one kg of inventory flow *i*. It must be mentioned that since waste marble is a by-product and no further processing is required, its environmental impact values were considered zero, as was similarly considered by Khodabakhshian et al. [53] and Khattab et al. [54].

However, due to additional processing required to reduce the size of the marble to be suitable for use as aggregate in concrete, a crushing stage was considered. This step entails energy consumption and associated carbon emissions. The crushing process was modeled based on a jaw crusher with an electrical power rating of 132 kWh and a throughput capacity of 120 tons per hour.

It is worth noting that for the preparation of marble powder, the fines used were directly sourced from the slurry generated during the stone-cutting process at the quarry. As this material was already in powder form, no secondary preparation steps were required, and it was treated as a zero-burden by-product with negligible additional environmental impact.

The energy required for the crushing operation was calculated using Equation (2), and the associated emissions were estimated using Equation (3) [55]:

(2) Energy consumption=power rating of equipment×operating hours

(3) E=A×EF×(1−ER/100)

As outlined by the U.S. Environmental Protection Agency (EPA), in the referenced emission calculation formula, *E* denotes the total emissions produced, *A* refers to the corresponding activity level (typically quantified as energy usage), *EF* indicates the emission factor, and *ER* reflects the percentage efficiency associated with emission reduction strategies. To maintain a conservative and robust assessment, the influence of the *ER* term was disregarded in line with the approach adopted by [55].

Given that Iran’s primary energy mix is predominantly reliant on crude oil, a conversion factor of 0.29 was applied to transform the final electricity consumption in kilowatt-hours (kWh) into its equivalent in kilograms of oil equivalent (kgoe). Additionally, to estimate carbon emissions from electricity consumption, a CO_2_ emission factor of 1.2 kg CO_2_eq per kgoe was used, as recommended by [56].

The environmental impacts from the transportation of raw materials—including energy use and carbon emissions—were estimated based on average diesel truck transport. An energy consumption rate of 3.2667 MJ/ton·km and an emission rate of 234.85 g CO_2_/ton·km were used for these calculations [57]. The materials used in this study were sourced from local suppliers with transport distances of approximately 50 km for cement, 10 km for sand, and 100 km for marble.

##### Overview of Results

**Figure 6:**
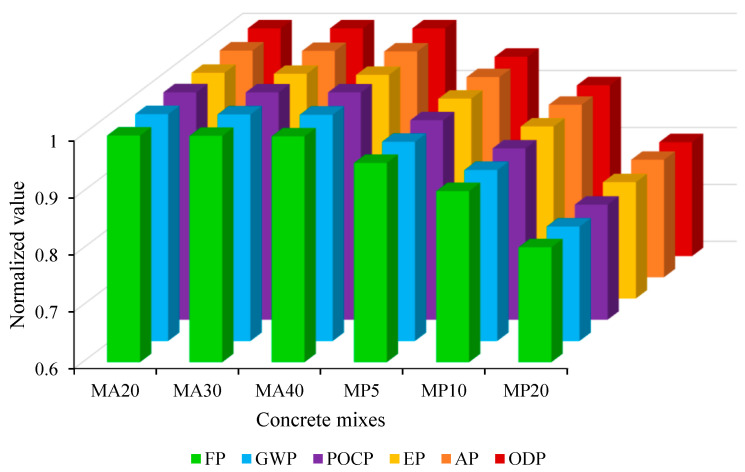
The environmental indicators for all concrete mixes.

This section first discusses the comparison of the above environmental indicators in all concrete samples. Figure 6 presents the normalized values of six key indicators—ODP, AP, EP, POCP, GWP, and FP—relative to the control mix to visually highlight the relative environmental performance of each mix incorporating WMP or WMA. Due to the normalization process and the relatively small changes in the proportions of cement and aggregates, the resulting indicator values appear close for most mixes. This graphical proximity reflects the limited variability in mass contributions from substituted materials, especially in the case of aggregate replacement. To enhance the clarity and interpretability of the results, Table 4 provides the actual (non-normalized) environmental indicator values for all mixes, including the control.

It was observed that using wastes instead of raw materials in concrete production has generally reduced its environmental impacts, as other studies have also presented this outcome [2,53,54].

The findings reveal that by a constant amount of cement and replacing part of the natural aggregate with WMA in concrete samples MA20, MA30, and MA40, a smaller reduction in the values of environmental indicators than CM compared to the concrete samples MP5 to MP20 was obtained. In this way, the highest variation was related to EP and then AP (reduction of about 0.7% and 0.3%, respectively). Considering the smaller portion of natural aggregate than cement in these indicators (according to Table 3), this trend of change is expected. Meanwhile, the environmental indicators for concrete mixes containing recycled marble aggregate as a substitute for natural aggregate are very close to each other. It is important to note that, for example, CO_2_ emissions (or GWP index) from the use of natural aggregates have a similar amount to emissions from the use of recycled aggregates in concrete [58]. Besides, it was concluded that increasing the amount of recycled marble aggregate led to a decrease in the environmental indicators, although there was a slight difference with the CM (using natural aggregate).

In the following, by a constant amount of natural aggregate and reducing the amount of cement (adding WMP), the values of environmental indicators and, thus, the negative environmental effects of concrete production are significantly reduced. According to Table 3, the value of the examined indicators for cement was much higher than other constituents of concrete. Therefore, any reduction in the amount of cement has a significant impact on the value of each environmental index for the concrete sample. In this way, by increasing the replacement of cement with marble powder from 5% to 20% (in samples MP5 and MP20), the reduction in environmental indicators compared to the CM has increased by about 5% to 20%. Belaidi et al. [59] also concluded that marble waste is more beneficial when used as a cement substitute rather than an aggregate, offering substantial reductions in environmental burdens such as carbon emissions.

**Table 3:** Life cycle inventory of concrete raw materials.

Indicator	ODP	AP	EP	POCP	GWP	FP
Unit	kg R11/kg	kg SO_2_/kg	kg PO_4_/kg	kg C_2_H_4_/kg	kg CO_2_/kg	MJ/kg
Cement	7.22 × 10^−9^	1.72 × 10^−3^	2.10 × 10^−4^	1.70 × 10^−4^	0.7634	4.727
Water	3.01 × 10^−11^	1.31 × 10^−4^	7.28 × 10^−7^	5.88 × 10^−8^	0.0025	0.00574
Natural fine agg.	0.00	9.58 × 10^−6^	2.49 × 10^−6^	1.25 × 10^−7^	0.0028	0.022
Superplasticizer	2.30 × 10^−10^	2.92 × 10^−3^	1.03 × 10^−3^	2.12 × 10^−3^	0.7670	18.3
Sources	[60]	[60]	[60]	[60]	[53,58]	[61]

**Table 4:** The environmental indicators for all concrete mixes.

Mix ID	ODP (kg R11/kg)	AP (kg SO_2_/kg)	EP (kg PO_4_/kg)	POCP (kg C_2_H_4_/kg)	GWP (kg CO_2_/kg)	FP (MJ/kg)
CO	0.000006796	1.6725158	0.2021187	0.1620291	707.63364	4397.725856
MP5	0.000006457	1.5916758	0.1922487	0.1540391	672.51724	4180.283856
MP10	0.000006117	1.5108358	0.1823787	0.1460491	637.40084	3962.841856
MP20	0.000005439	1.3491558	0.1626387	0.1300691	567.16804	3527.957856
MA20	0.000006796	1.6698142	0.2014165	0.1619939	706.86084	4391.653856
MA30	0.000006796	1.6684635	0.2010654	0.1619763	706.47444	4388.617856
MA40	0.000006796	1.6671127	0.2007143	0.1619586	706.08804	4385.581856

**Figure 7:**
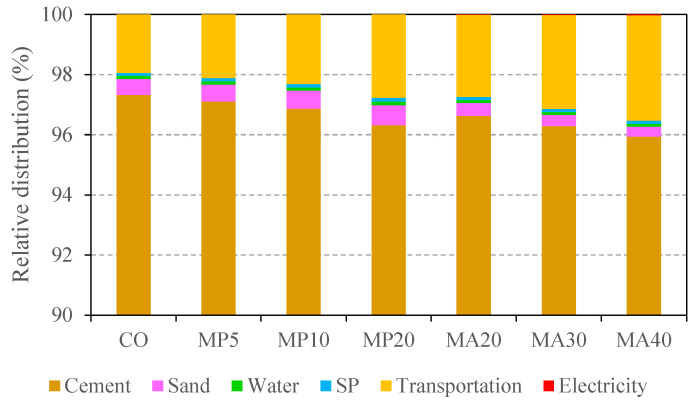
Relative distribution of GWP for concrete mixes across material production, transportation, and processing stages.

**Figure 8:**
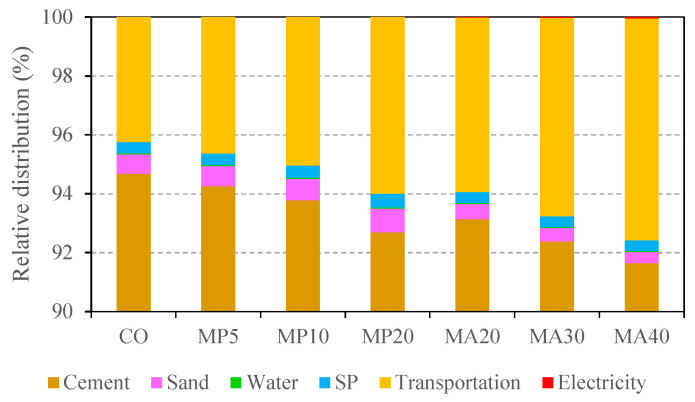
Relative distribution of energy consumption for concrete mixes across material production, transportation, and processing stages.

The environmental analysis thus far has focused on the raw material production phase. To provide a more comprehensive view, Figure 7 and Figure 8 present the relative contributions of individual components to total GWP and energy consumption, including production, transportation, and processing stages.

Figure 7 illustrates the proportional GWP contributions (in kg CO_2_/kg) of cement, sand, water, superplasticizer (SP), transportation of all constituents (including marble waste), and the energy consumed during aggregate crushing. Cement production clearly dominates, accounting for roughly 96–97% of the total GWP across all mixes, reaffirming the critical importance of its reduction via WMP incorporation. Transportation follows with a 2–3.5% share, while other materials contribute minimally.

Figure 8 shows the relative distribution of total energy consumption, considering primary material production, transportation, and processing stages. Once again, cement accounts for the largest share—approximately 92% to 95%—of total energy use during raw material production. As marble waste replacement increases, energy use associated with transportation rises (up to 7.5%). In contrast, sand and SP contribute less than 0.8% and 0.4%, respectively. Notably, energy consumption during marble aggregate crushing was negligible compared to the other stages.

#### 4.2.2. Eco-Cost-Mechanical Assessment

The sustainability potential of concrete mixtures is closely linked to a combination of mechanical performance, durability, environmental footprint, and cost efficiency. The introduction of recycled and waste materials, such as WMP and WMA, not only influences technical properties but also impacts economic viability and ecological outcomes. To quantify the economic component of each concrete mix in this study, the cost was calculated using the method adapted from [62]. The cost of constituent materials was determined based on the unit prices listed in Table 5.

**Figure 9:**
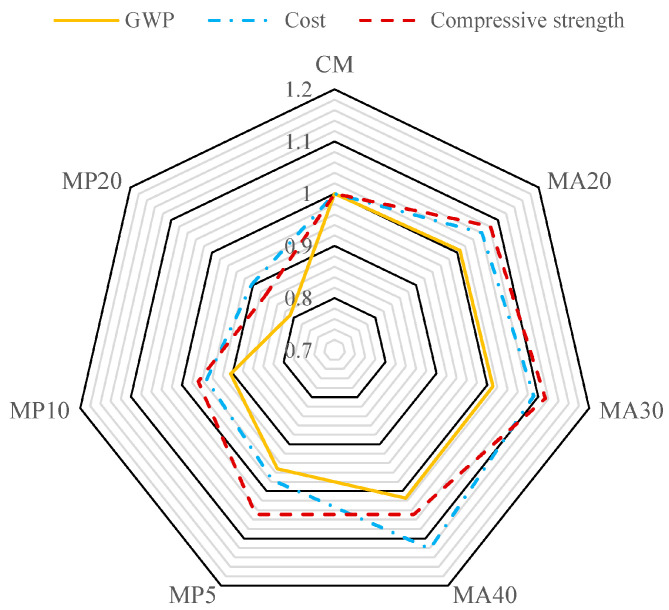
Combination of normalized mechanical, economic, and environmental indicators.

This evaluation considered three primary indicators to represent sustainability dimensions: compressive strength (mechanical), GWP (environmental), and calculated cost (economic). To allow direct comparison, all values were normalized relative to the CM. The results, presented in Figure 9, highlight performance variations among the tested mixtures.

From a mechanical perspective, the mixes incorporating WMA performed favorably, with MA30 achieving the highest strength enhancement, a gain of approximately 11% over the CM due to 30% marble aggregate replacement. MP5, incorporating 5% WMP, also improved strength by 4.9%. Furthermore, all mixes containing marble waste exhibited improved acid resistance compared to the control, reinforcing their suitability for harsh environments.

As shown in Figure 9, for concrete mixes incorporating WMP, increasing the cement replacement level from 5% to 20% led to a reduction in the GWP by approximately 5% to 20% relative to the CM. This trend confirms the significant role of cement in contributing to the environmental footprint of concrete, particularly CO_2_ emissions. In contrast, the incorporation of WMA as a partial replacement for natural aggregate had a negligible impact on the GWP, indicating that aggregate substitution contributes less to emission reduction.

Moreover, the cost analysis of the concrete mixtures revealed that replacing cement with WMP is more economically advantageous than substituting natural aggregate with WMA. Specifically, mixes containing WMA exhibited a cost increase ranging from approximately 6% (MA20) to 12% (MA40) compared to the control mix. Conversely, using WMP as a partial cement substitute led to cost reductions of about 3% in MP5 and up to 10% in MP20, demonstrating the economic efficiency of WMP integration.

#### 4.2.3. Optimization of Triple Indicators

Optimization serves as a critical method for identifying the most effective design solution, particularly in contexts requiring balanced decision-making. This approach involves the formulation of objective functions (performance criteria), the selection of independent variables (e.g., material proportions), and the assessment of dependent variables (e.g., resulting properties) under specific constraints [63]. In concrete mixture optimization, key performance targets may include strength, durability, cost-effectiveness, or environmental impact. The dependent responses, derived from varying input parameters, are benchmarked against these performance goals. In the present study, desirability functions were applied based on Equations (4) and (5) to convert the response values into dimensionless scores ranging from 0 (least desirable) to 1 (most desirable), as outlined in [64]. Equation (4) was used for indicators where lower values are more favorable, such as cost and environmental metrics (e.g., GWP), while Equation (5) was applied to indicators where higher values are preferred, such as compressive strength.

(4) $$d_{j}={\left(\frac{{maxf}_{j}-Y_{j}}{{maxf}_{j}-{minf}_{j}}\right)}^{t_{j}}$$

(5) $$d_{j}={\left(\frac{Y_{j}-{minf}_{j}}{{maxf}_{j}-{minf}_{j}}\right)}^{t_{j}}$$

where $Y_{j}$: current response; ${maxf}_{j}$: the highest value of the *j*th response; ${minf}_{j}$: the lowest value of the *j*th response; $t_{j}$: the weighting factor of the *j*th response; $d_{j}$: individual desirability function. It should be noted that $t_{j}$ factor was considered equal to 1 due to the same importance of the independent responses [65].

To identify the optimal concrete mix, a multi-response optimization strategy was employed. The composite desirability score (*D*) was determined by aggregating the individual desirability indices, as per Equation (6), where a *D*-value closer to 1 indicates a more optimal solution [7].

(6) $$D=\sqrt[{m}]{d_{1}\times d_{2}\times \ldots \times d_{m}}$$

where *m*: number of responses. Table 6 provides the individual and overall desirability functions for all concrete mixes. In that, *d*_1_ to *d*_5_ refer to environmental indicators, including AP, EP, POCP, GWP, and energy consumption, respectively. It is noteworthy that the total values of both GWP and energy consumption were calculated by accounting for all stages, including material production, material preparation, and transportation. The function d_6_ is related to the production cost of concrete. Next, *d*_7_ to *d*_11_ state the mechanical properties of concrete samples as follows. CS is compressive strength without sulfuric acid attack; CS-28 and CS-63 are compressive strength after 28 and 63 days of acid immersion; Mass-28 and Mass-63 are the percentage of residual mass after 28 and 63 days of acid immersion.

According to the calculation of d for each index in different mixtures, its value for ODP in MA20, MA30, and MA40 mixing plans was obtained as zero, resulting in the inability to properly compare with zero D. Therefore, the value of D was calculated again without considering ODP. The results for the D values were then compared and analyzed over all concrete mixes (presented in Table 6). Nevertheless, the highest value of *D* was assigned to the MP10 concrete sample. In that, the amount of aggregate did not change compared to the CM, but 10% of the cement was replaced with WMP. However, it was expected that the MP20 sample with 20% replacement of cement with WMP would have the highest *D* value by reducing the negative environmental effects of cement. This sample has the lowest value of compressive strength, and according to Equation (5) with *d* = 0, it has led to *D* becoming zero. Therefore, the MP10 sample has the highest overall desirability value considering the three aspects: mechanical, economic, and environmental aspects.

## 5. Conclusions

This study evaluated the mechanical performance, acid resistance, environmental impacts, and economic viability of SCC incorporating WMP as a partial cement replacement and WMAs as a partial fine aggregate replacement. The following conclusions can be drawn:

1. Mechanical performance: the optimal mechanical performance was achieved with 5% cement replacement by WMP, where improved matrix densification and particle packing enhanced compressive strength. However, higher WMP levels (10–20%) led to strength reductions due to dilution of reactive binders. In contrast, replacing natural sand with WMA at 20–40% consistently improved strength—peaking at 30% with an 11.5% increase—driven by superior particle grading, enhanced packing density, and improved interfacial transition zone characteristics.
2. Durability in acidic environments: all SCC mixtures incorporating marble waste exhibited enhanced resistance to sulfuric acid exposure relative to the control. The MP20 and MA40 mixes showed the least mass and strength losses after 63 days, indicating superior chemical durability. This improvement is attributed to the formation of a temporary protective gypsum layer from the reaction between calcium carbonate and sulfuric acid, which helped slow surface degradation and maintain structural integrity.
3. Environmental and economic benefits: The results demonstrated that replacing cement with WMP significantly reduced the GWP by 5% to 20% for substitution levels of 5% to 20%, highlighting the dominant role of cement in concrete’s environmental footprint. In contrast, the use of WMA as a partial sand replacement had minimal effect on GWP. From an economic standpoint, WMP substitution also proved more beneficial, reducing production costs by up to 10%, whereas mixes with WMA showed increased costs of up to 12% compared to the control. These findings confirm the environmental and economic advantages of WMP over WMA in sustainable SCC design.
4. Integrated sustainability optimization: multi-criteria optimization using desirability functions identified MP10 (10% WMP) as the most balanced and sustainable mix, effectively integrating strength, durability, environmental, and economic performance. Although MP20 offered the highest environmental gain, its compromised mechanical strength limited its structural applicability.

The findings strongly support the reuse of marble industry waste in SCC, offering enhanced durability, reduced environmental impact, and cost savings, particularly valuable in aggressive environments such as sewage and wastewater facilities. However, this study did not include microstructural evidence (e.g., SEM, XRD, porosity), which limits the mechanistic interpretation of phenomena such as the filler effect and acid resistance. These aspects should be explored in future research.

Future research is recommended to:

- Explore combined replacement strategies involving both WMP and WMA (e.g., MP5MA20, MP10MA30) to assess potential synergistic enhancements in mechanical, durability, and environmental performance;
- Investigate the integration of marble waste with supplementary industrial materials such as fly ash, ground granulated blast furnace slag, or ceramic waste to optimize binder efficiency and sustainability;
- Assess the long-term performance and durability of marble waste-incorporated SCC under realistic environmental exposures, including variable temperature, humidity, and aggressive chemical conditions;
- Extend the application of marble waste into advanced cementitious systems, including high-performance concrete, ultra-high-performance concrete, and alkali-activated/geopolymer matrices;
- Employ advanced microstructural characterization techniques (e.g., SEM, XRD, MIP, FTIR) to elucidate the underlying mechanisms governing strength development, durability enhancement, and chemical resistance.

Such research directions would not only reinforce the mechanistic understanding of marble waste incorporation but also expand its practical deployment in sustainable, high-performance concrete solutions.

## Figures and Tables

**Table 1:** Chemical characteristics of the OPC and waste marble.

Chemical Compound (%)	SiO_2_	Al_2_O_3_	Fe_2_O_3_	CaO	MgO	Na_2_O	SO_3_	Loss of Ignition
OPC	20.97	4.37	3.74	63.21	1.77	0.38	2.05	1.1
WM	0.46	0.05	0.32	53.49	0.12	0.01	0.015	43.26 *

* The high LOI value (43.26%) for WM powder is primarily due to the decomposition of calcium carbonate (CaCO_3_) present in marble, resulting in the release of CO_2_ upon heating during the LOI determination.

**Table 2:** Concrete mix proportions.

Specimen	Designation of Mixes	Binder		Fine Aggregate	
		OPC (%)	WMP (%)	WMA (%)	Sand (%)
Control	CM	100	0	0	100
Series-I	MP5	95	5	0	100
	MP10	90	10	0	100
	MP20	80	20	0	100
Series-II	MA20	100	0	20	80
	MA30	100	0	30	70
	MA40	100	0	40	60

**Table 5:** Cost of ingredients.

Material	Cement	Aggregate	SP	Water	WMA	WMP
Cost (Euro/kg)	0.021	0.053	0.10	0.82	0.02	0.10
Sources	[62]	[62]	[62]	[62]	*	*

* Costs were determined by the authors.

**Table 6:** Individual and overall desirability functions for concrete mixes.

Mix ID	Individual Desirability Function											Overall Desirability Function (*D*)
	*d*_1_	*d*_2_	*d*_3_	*d*_4_	*d*_5_	*d*_6_	*d*_7_	*d*_8_	*d*_9_	*d*_10_	*d*_11_	
	AP	EP	POCP	GWP	Energy	Cost	CS	CS-28	CS-63	Mass-28	Mass-63	
CM	0.000	0.000	0.000	0.071	0.157	0.556	0.588	0.368	0.064	0.000	0.000	0.000
MP5	0.252	0.250	0.250	0.303	0.367	0.667	0.765	0.639	0.544	0.736	0.352	0.422
MP10	0.504	0.500	0.500	0.536	0.578	0.778	0.471	0.333	0.224	0.434	0.816	0.476
MP20	1.008	1.000	1.000	1.000	1.000	1.000	0.000	0.000	0.000	0.811	1.000	0.000
MA20	0.008	0.018	0.001	0.036	0.078	0.278	0.882	0.806	0.816	0.547	0.572	0.107
MA30	0.013	0.027	0.002	0.018	0.039	0.139	1.000	1.000	1.000	0.736	0.851	0.111
MA40	0.017	0.036	0.002	0.000	0.000	0.000	0.765	0.771	0.832	1.000	0.931	0.000

## Data Availability

The original contributions presented in this study are included in the article. Further inquiries can be directed to the corresponding author.
